# Supplementary material for: Identification of photoperiod-induced specific miRNAs in the adrenal glands of Sunite sheep (Ovis aries)
Source: Front Vet Sci. 2022 Jul 22;9:888207. doi: 10.3389/fvets.2022.888207 (PMC9354845; doi:10.3389/fvets.2022.888207)
Supplement: Supplementary file 1 [file Data_Sheet_1.ZIP › Supplementary file/miRDeep2 file.zip!/miRDeep2 file/Novel_Structure/SPLP42A1/Novel_30.pdf]

Provisional ID	:	NC_019484.2_32051
Score total	:	115.5
Score for star read(s)	:	3.9
Score for read counts	:	107.7
Score for mfe	:	2.3
Score for randfold	:	1.6
Score for cons. seed	:	
Total read count	:	223
Mature read count	:	215
Loop read count	:	0
Star read count	:	8

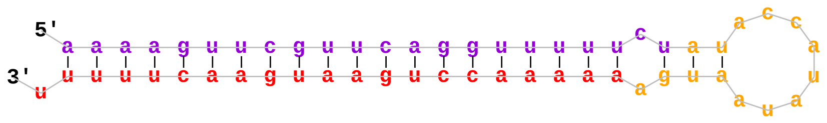

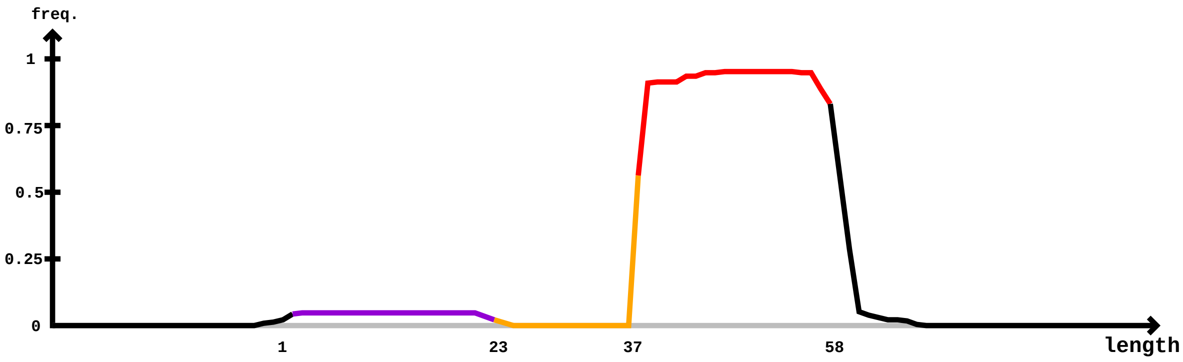

Star **Mature**

```
5'- uuccauaaauacuggguugggcaaaaaguucguucagguuuuucuauaccauauaaugaaaaaaccugaaugaacuuuuuggccaacccuguauuuuccuugguggcugcaaa   -3'   obs
    uuccauaaauacuggguugggcaaaaaguucguucagguuuuucuauaccauauaaugaaaaaaccugaaugaacuuuuuggccaacccuguauuuuccuugguggcugcaaa         exp
    ..(((((((((.(((((((.(((((((((((((((((((((((.(((........))).))))))))))))))))))))))).))))))).))))))......))))....... reads   mm   sample
    ....................gcaaaaaguucguucagguuuuu...................................................................... 2   0   seq
    .....................caaaaaguucguucGgguuuuu...................................................................... 1   1   seq
    ......................aaaaaguucguucGgguuuuuc..................................................................... 2   1   seq
    .......................aaaaguucguucGgguuuuucu.................................................................... 5   1   seq
    ........................aaaguucguucaggCuuuuc..................................................................... 1   1   seq
    ...........................................................aaaaaccugaaugaacC..................................... 1   1   seq
    ...........................................................aaaaaccugaaugaacuuu................................... 1   0   seq
    ...........................................................aaaaaUcugaaugaacuuu................................... 9   1   seq
    ...........................................................aaaaaccugaaGgaacuuu................................... 1   1   seq
    ...........................................................aaaaaccAgaaugaacuuu................................... 1   1   seq
    ...........................................................Gaaaaccugaaugaacuuuu.................................. 1   1   seq
    ...........................................................aaaaaccugaaugaacuuuu.................................. 1   0   seq
    ...........................................................aGaaaccugaaugaacuuuu.................................. 1   1   seq
    ...........................................................aaaaaccAgaaugaacuuuu.................................. 7   1   seq
    ...........................................................aaaaaUcugaaugaacuuuuu................................. 80  1   seq
    ...........................................................Caaaaccugaaugaacuuuuu................................. 1   1   seq
    ...........................................................aaaaaccugGaugaacuuuuu................................. 1   1   seq
    ...........................................................aaaaaccugaaugaacuuuuC................................. 2   1   seq
    ...........................................................Gaaaaccugaaugaacuuuuu................................. 1   1   seq
    ...........................................................aaaaaccugaGugaacuuuuu................................. 1   1   seq
    ...........................................................aaaaaccAgaaugaacuuuuu................................. 1   1   seq
    ...........................................................aaaaaccAgaaugaacuuuuug................................ 3   1   seq
    ...........................................................Gaaaaccugaaugaacuuuuug................................ 1   1   seq
    ...........................................................aaaaaccugaaugaacCuuuug................................ 1   1   seq
    ...........................................................aaaaaccugaaugaacuuuuuA................................ 4   1   seq
    ...........................................................aaaaaccugaaGgaacuuuuug................................ 8   1   seq
    ...........................................................aaaaaccugaaugaacuuuuugU............................... 2   1   seq
    ...........................................................aaaaaccugaaugaacuuuuuUg............................... 1   1   seq
    ............................................................aaaaccAgaaugaacuuu................................... 1   1   seq
    ............................................................aaaaccugaaugaacuuu................................... 1   0   seq
    ............................................................aaaaccAgaaugaacuuuu.................................. 1   1   seq
    ............................................................aaaaccugaaugaacuuuu.................................. 1   0   seq
    ............................................................aaaaccugGaugaacuuuu.................................. 1   1   seq
```

```
                       Star                                Mature
uuccauaaauacuggguugggcaaaaaguucguucagguuuuucuauaccauauaaugaaaaaaccugaaugaacuuuuuggccaacccuguauuuuccuugguggcugcaaa
............................................................Gaaaccugaaugaacuuuuu.................................   1    1    seq
............................................................aaaaccugaaugaacuuuuu.................................   11   0    seq
............................................................aaaaccugaaugaacCuuuu.................................   1    1    seq
............................................................aaaaccGgaaugaacuuuuu.................................   1    1    seq
............................................................aaaaccAgaaugaacuuuuu.................................   3    1    seq
............................................................aaaaccugaaugaacuuuuuA................................   1    1    seq
............................................................aaaaccugaaugaacCuuuug................................   3    1    seq
............................................................aaaaccugaaugaacuuuuugg...............................   34   0    seq
............................................................aaaaccugaaugaacCuuuugg...............................   5    1    seq
............................................................aaaaAcugaaugaacuuuuugg...............................   1    1    seq
............................................................aaaaccugaaugaacuuuuugU...............................   1    1    seq
............................................................aaaaccugaaugaacuuuuugA...............................   10   1    seq
............................................................aaaaccugaaugaacuuuuuggU..............................   1    1    seq
............................................................aaaaUcugaaugaacuuuuuggc..............................   2    1    seq
.............................................................aaaccGgaaugaacuuuuug................................   1    1    seq
................................................................ccugaaugaacuuuuuggcc.............................   2    0    seq
................................................................ccugaaugaacuuuuuggccC............................   1    1    seq
................................................................ccugaaugaacuuuuuggcca............................   1    0    seq
................................................................ccugaaugaacuuuuuggccaUc..........................   1    1    seq
..................................................................ugaaugaacuuuuuggccaacc.........................   1    0    seq
..................................................................ugaaugaacuuuuuggccaacA.........................   1    1    seq
..................................................................ugaaugaacuuuuuggUcaaccc........................   1    1    seq
....................................................................aaugaacuuuuuggccaacc.........................   1    0    seq
```
